# The influence of the host on the course of gastric carcinoma.

**DOI:** 10.1038/bjc.1992.376

**Published:** 1992-11

**Authors:** C. W. Janssen, R. T. Lie, C. F. Bassøe, H. Maartmann-Moe, R. Matre

**Affiliations:** Department of Surgery, Gade Institute, University of Bergen, Norway.

## Abstract

Immunoglobulins (Ig) and some complement components (C) were quantified in sera from patients with gastric carcinoma before surgery and at regular intervals during a 5-year follow-up. The preoperative concentrations of C1-INH and C4 were higher (P < 0.0005 and P < 0.005) and IgG lower (P < 0.0005) in 50 patients with recurrence than in 46 5-year survivors. The prognostic significant of C1-INH was superior to that of the extent of disease (F-values 37.1 and 26.1). The preoperative immune data classified 76% of the patients correctly as to recurrence and no recurrence. Also, the preoperative C1-INH concentration had a highly significant effect on time to recurrence of cancer (P = 0.0007), adjusting for age and disease extent. After surgery the mean IgG concentrations were within normal range and without difference between the two groups. On the other hand, the concentrations of C1-INH and C4 in the individual patients in both groups remained the same from before to after surgery and throughout the observation period (P = 0.34). Apparently, the serum levels of C1-INH and C4 do not reflect the bearing of cancer. We therefore suggest that these variables represent an independent immune state that is appropriate to the host. A comparison of our variables with those of healthy individuals seems to support this idea. This immune state has a significant influence on whether a resected gastric cancer will recur, and also on how soon recurrence may be manifest.


Br. J. Cancer (1992), 66, 870-874 © Macmillan Press Ltd., 1992

### The influence of the host on the course of gastric carcinoma

C.W. Janssen Jr[1], R.T. Lie[2], C.-F. Bassøe[3], H. Maartmann-Moe[4] & R. Matre[5]

[1]*Department of Surgery,* [2]*Section for Medical Informatics and Statistics,* [4]*Department of Pathology and* [5]*Department of Microbiology and Immunology, The Gade Institute, University of Bergen;* [3]*General Practitioner, Bergen, Norway.*

**Summary** Immunoglobulins (Ig) and some complement components (C) were quantified in sera from patients with gastric carcinoma before surgery and at regular intervals during a 5-year follow-up.

The preoperative concentrations of C1-INH and C4 were higher ($P<0.0005$ and $P<0.005$) and IgG lower ($P<0.0005$) in 50 patients with recurrence than in 46 5-year survivors. The prognostic significant of C1-INH was superior to that of the extent of disease (F-values 37.1 and 26.1). The preoperative immune data classified 76% of the patients correctly as to recurrence and no recurrence. Also, the preoperative C1-INH concentration had a highly significant effect on time to recurrence of cancer ($P = 0.0007$), adjusting for age and disease extent.

After surgery the mean IgG concentrations were within normal range and without difference between the two groups. On the other hand, the concentrations of C1-INH and C4 in the individual patients in both groups remained the same from before to after surgery and throughout the observation period ($P = 0.34$).

Apparently, the serum levels of C1-INH and C4 do not reflect the bearing of cancer. We therefore suggest that these variables represent an independent immune state that is appropriate to the host. A comparison of our variables with those of healthy individuals seems to support this idea. This immune state has a significant influence on whether a resected gastric cancer will recur, and also on how soon recurrence may be manifest.

Is the altered immune state found in many cancer patients a consequence of malignancy or rather a state that influences the growth and spread of cancer? Traditionally, the main prognosticators of most cancers are the extent of disease at the time of treatment and the surgical technique. The long-term outcome within identical groups of patients may nevertheless be widely different, indicating that recurrence of cancer is regulated by one or more additional factors (Poste & Fidler, 1980). There is substantial evidence that the proliferation of cancer cells *in vitro* is affected by various immune mechanisms (Woodruff, 1982). On the other hand, the effect of the host's immune state on the growth of cancer *in vivo* is not so apparent.

We have studied patients with gastric carcinoma in a cumulative series starting in 1977. Some humoral immune factors in sera were quantified preoperatively and at regular intervals during a 5-year follow-up. Recently we showed that the preoperative levels of IgG and the complement components C1-inhibitor (C1-INH) and C4 were essential prognosticators of gastric carcinoma and that they were independent of the extent of disease (Janssen *et al.*, 1989).

### Materials amd methods

### *Patients*

A total of 143 patients with curative intent resection of gastric carcinoma in the Department of Surgery during the years 1977-84 were included in this study. Excluded were patients with other diseases or a history of another malignant disease within 5 years before gastric cancer surgery. The mean age (± 1 s.d.) was 67.1 ± 11.2 (range 31-87) years, median age was 68.0 years, and 38.3% were women. Adjunctive cancer therapy was not administered.

After surgery the patients entered a regular follow-up programme with examinations scheduled at every 3 months in the first year, later at 6-month intervals. The status of the series 5 years after surgery is shown in Table I.

During the first 5 years after surgery nine patients had a second primary cancer. Colon cancer appeared in four, urogenital cancers also in four and squameous cell lung cancer in one patient.

Twelve patients died from various causes without evidence of malignant disease at clinical or post-mortem (three patients) examination. One of them died from complications after a later laparotomy whereas 11 patients died from cardiopulmonary or cerebrovascular diseases.

Three patients were lost on follow-up. They are all dead; at the last follow-up examination 1-3 months before death they were without signs of malignancy.

Five years after surgery 46 patients were alive and without clinical signs of disease. During follow-up 50 patients had a clinical course consistent with recurrence of gastric carcinoma. These two groups of patients were compared to each other and to healthy individuals.

The median time between surgery and clinical signs of recurrence was 12.3 (range 2-59) months and the median time between signs of recurrence and death was 2.5 (range 0-34) months.

The age difference between the patients with and without recurrence was small and insignificant ($P>0.05$), and there was no significant sex difference between the groups ($P>0.6$). There was also no difference in the surgical procedure for total gastrectomy *vs* less extensive procedures ($P<0.2$) or splenectomy *vs* no splenectomy ($P>0.4$).

### *Healthy individuals*

One hundred and ten consecutive healthy individuals from an annual medical check-up was also used in the study. Individuals with chronic diseases or a history of cancer were ex-

**Table I** Survey of 143 patients 5 years after potentially curative surgery for gastric carcinoma (1977-1984)

| | *No. of patients* |
|---|---|
| Alive and clinically cancer-free | 46 |
| Clinical course consistent with recurrence | 50 |
| Cancer in the vicinity of the resection border | 11 |
| Died in hospital after the operation | 12 |
| Died later from various causes without evidence of malignant disease | 12 |
| Second primary cancer after gastric cancer surgery | 9 |
| Lost to follow-up | 3 |

Correspondence: C.W. Janssen Jr, Department of Surgery, Haukeland University Hospital, N-5021 Bergen, Norway.
Received 23 April 1991; and in revised form 18 June 1992.

cluded. Also excluded were those with an acute disease at the time of check-up or during the preceding months. The mean age ( ± 1 s.d.) was 44.6 ± 14.5 (range 20–78) years and 35% were women.

### Pathology

Based on the criteria advised by IUCC (Hermanek & Sobin, 1987), the cancer patients were divided into six groups of extent of disease at the time of surgery; T1N0M0, T2N0M0, T3N0M0, T2N + M0, T3N + M0, T4NXM0 (NX, i.e. irrespective of lymph nodes). One patient with T1N + M0 disease was assigned to the T2N + M0 group. No patients with distant metastases underwent potentially curative surgery.

### Sera

Concentrations of IgG, IgA and IgM and the complement components C3, C4 and C1-INH were quantified in sera as previously described (Janssen *et al.*, 1987*a*).

Preoperative blood samples were missing in ten patients, either because gastric ulcer was the primary diagnosis at the time of surgery or because they were operated shortly after admission and before blood was sampled for quantification of Ig and C.

### Statistics

Mean values were compared by Student's *t*-test, preceded by Fisher's test for comparison of variances. Distributions of nominal data were tested in $\chi^2$ tests with Yates' modification. The distribution of + and − signs of the differences in serial observations was tested by the sign test (Lentner, 1982).

The predictive value of the preoperative variables was assessed by discriminant analysis, performed by the program 7M in BMDP (Jennrich & Sampson, 1990). The groups of disease extent were ranged in the order 1–6 and entered the discriminant analysis as an independent variable. The effect of the variables on time interval from surgery to recurrence was also tested in a Cox proportional hazard regression analysis using the program 2L in BMDP (Hopkins, 1990). Differences between groups of patients in the profile of pre- and postoperative values of C1-INH were tested in analysis of variance with repeated measures. The program 5V in BMDP was used because of an unbalanced design and incomplete data (Schluchter, 1990).

### Results

### Preoperative data and extent of disease

The mean preoperative serum concentration of IgG was lower and the concentrations of C4 and C1-INH higher in patients with recurrence of gastric carcinoma as compared to the patients alive and disease-free 5 years after surgery (Table II). The concentration of IgA, IgM and C3 were not different between the groups.

Discriminant analysis of the preoperative immune variables classified 76% of the patients correctly as to recurrence. When the extent of disease, grouped in the order 1–6, entered the analysis as an independent variable, the correct classification was 83% (Jackknifed classification). C1-INH was the most potent discriminator, followed by extent of disease, IgG and C4 in that order of significance, Table III. In addition to the discriminating potential of C1-INH the potential of disease extent was considerably less and IgG and C4 were insignificant. This result was consistent with the result of a proportional hazard regression analysis using time to recurrence.

The patients were divided into six groups of C1-INH concentrations. The prognosis of patients in each group was compared to that of the six groups of disease extent, Figure 1. The risk for recurrence within 5 years after surgery increased evenly both with increasing C1-INH and increasing extent of disease. In the group of patients with the highest C1-INH ( $\geqslant 0.50\ \mathrm{gl^{-1}}$), the recurrence rate was 1.0 as opposed to 0.78 among the patients with the most advanced disease.

The patients with recurrence were arbitrarily divided into three groups, those with recurrence $\leqslant 8$ months, 9–17 months and $\geqslant 18$ months. The preoperative C1-INH concentration was higher where the time interval from surgery to clinical signs of recurrence was shorter, Figure 2. Moreover, a proportional hazard regression analysis showed that the preoperative C1-INH concentration had a highly significant effect on time to recurrence of cancer ($P = 0.0007$), adjusting for age and disease extent. The mean preoperative values in the three groups of patients with recurrence were 0.48, 0.44 and 0.41 $\mathrm{gl^{-1}}$ respectively (and 0.35 $\mathrm{gl^{-1}}$ for the 5 year survivors).

On the other hand, there was no clear pattern in the relation between the groups of disease extent and the time interval from surgery to signs of recurrence. The median time interval was at most 18.0 months in the T3N0 group and at least 10.5 months in the T3N + group.

The preoperative data of the patients dying from various causes without signs of cancer and of the patients with cancer at the resection border were compared to those of the patients with and without recurrence. The values could not be assigned to any of the two groups (Gamel *et al.*, 1986).

### Postoperative data

The mean concentrations of C1-INH and C4 in the groups of patients with and without recurrence were the same 3 and 6 months after surgery as before surgery, Table IV. The concentrations of C1-INH and C4 preoperatively and 3 months postoperatively in the individual patients were compared by the sign test, which was insignificant ($P > 0.2$). Furthermore, whenever the first postoperative C1-INH recording was $\geqslant 0.45\ \mathrm{gl^{-1}}$, the recurrence rate was 1.0.

The pre- and postoperative C1-INH in the three groups of patients with recurrence $\leqslant 8$ months, 9–17 months and $\geqslant 18$ months were compared in an anlaysis of variance model with

**Table II** Preoperative serum concentrations ($\mathrm{gl^{-1}}$, mean ± 1 s.d.) of immunoglobulins and complement components in 43 patients with recurrence and in 43 patients alive and clinically disease-free 5 years after potentially curative surgery for gastric carcinoma

| | *Cancer recurrence* | *No recurrence* | *Significance of differences* |
|---|---|---|---|
| IgG | 8.91 ± 2.43 | 11.28 ± 3.57[a] | $P < 0.0005$ |
| IgA | 2.35 ± 1.18 | 2.25 ± 1.14 | n.s. |
| IgM | 1.35 ± 1.44 | 1.20 ± 0.61[a] | n.s. |
| C3 | 0.90 ± 0.24 | 0.86 ± 0.15[a] | n.s. |
| C4 | 0.43 ± 0.12 | 0.37 ± 0.11 | $0.005 > P > 0.001$ |
| C1-INH | 0.43 ± 0.07 | 0.35 ± 0.07 | $P < 0.0005$ |

[a]Inhomogeneity of variances, Welch's correction applied (Diem & Lentner, 1982).

**Table III** The potential (as F-values) of disease extent (ordered in groups 1–6) and the preoperative concentrations of IgG, C4 and C1-INH to discriminate between patients with and without recurrence after potentially curative surgery

| *Variable* | *F-value* | *Statistical significance* |
|---|---|---|
| C1-INH | 37.07 | $P < 0.001$ |
| Disease extent | 26.08 | $P < 0.001$ |
| IgG | 14.61 | $P < 0.001$ |
| C4 | 6.91 | $0.025 > P > 0.01$ |

Number of patients: 86.

**Figure 1** The risk for recurrence withint 5 years after potentially curative surgery for gastric carcinoma; **a**, in groups of preoperative Cl-INH concentration; **b**, in groups of disease extent.

**Figure 2** The mean serum Cl-INH concentrations ($g\,l^{-1}$) before and after surgery in patients with gastric carcinoma, divided into groups as indicated in the figure. No manifest recurrence at the time of sampling.

repeated measurements. The between group difference was highly significant ($P<0.0001$, between factor). Observations of clinically disease-free individuals in the respective groups were few beyond those indicated in Figure 2. Apparently, Cl-INH levels remained unchanged from before to after surgery and throughout the observation period ($P=0.34$, within factor). Among the long time survivors the mean Cl-INH concentrations increased slightly and evenly during the 5-year observation time (Figure 2).

In addition, the postoperative serum concentrations of C4 in each of the three groups of patients with recurrence and among the long time survivors stayed much at the same levels as before surgery. There were, however, some variations at each time interval in the recurrence groups.

Interestingly, discriminant analysis comprising Cl-INH and C4 as recorded 6 months after surgery classified 71% of the patients correctly in the recurrence and no recurrence groups.

In both groups of patients the mean IgG concentrations were within the normal range after surgery and throughout the observation period, with no difference between the two groups. The postoperative concentrations of IgG were independent of the preoperative recordings (Table IV).

*Healthy individuals*

The concentrations of Ig and C are presented in Table V. There was no difference between the sexes. Some variables were age dependent as indicated. The variations with age

**Table IV** Serum concentrations ($gl^{-1}$, mean ± 1 s.d.) of IgG, C4 and Cl-INH before and 3 and 6 months after surgery in patients with and without recurrence within 5 years after surgery

| | | *Preop.* | *Diff.* | *Three months postop.* | *Diff.* | *Six months postop.* |
|---|---|---|---|---|---|---|
| | | $n=43$ | | $n=26$ | | $n=27$ |
| No | IgG | 11.28±3.57 | $P<0.001$ | 14.96±4.23 | n.s. | 14.49±3.41 |
| recurrence | C4 | 0.37±0.11 | n.s. | 0.35±0.10 | n.s. | 0.34±0.08 |
| | Cl-INH | 0.35±0.07 | n.s. | 0.35±0.05 | n.s. | 0.35±0.07 |
| With | | $n=43$ | | $n=31$ | | $n=24$ |
| recurrence | IgG | 8.91±2.43 | $P<0.001$ | 14.56±3.67 | n.s. | 13.84±3.14 |
| | C4 | 0.43±0.12 | n.s. | 0.42±0.11 | n.s. | 0.41±0.11 |
| | Cl-INH | 0.43±0.07 | n.s. | 0.41±0.10 | n.s. | 0.43±0.10 |

N.B. no manifest recurrence at the time of sampling.

**Table V** Serum concentrations of immunoglobulins and complement components in 110 healthy individuals and the variation with age

| *Variable* | *Mean ± 1 s.d.* | *Coefficient of regression with age* | *P-value* |
|---|---|---|---|
| IgG $g\,l^{-1}$ | 12.73 ± 2.49 | −0.0207 | n.s. |
| IgM $g\,l^{-1}$ | 2.51 ± 1.00 | 0.0148 | 0.0464 |
| IgA $g\,l^{-1}$ | 1.33 ± 0.61 | −0.0108 | 0.0186 |
| C3 $g\,l^{-1}$ | 0.74 ± 0.12 | 0.0002 | n.s. |
| C4 $g\,l^{-1}$ | 0.35 ± 0.11 | 0.0017 | 0.0390 |
| C1-INH $g\,l^{-1}$ | 0.35 ± 0.06 | 0.0025 | <0.0001 |

Mean age of the individuals ± 1 s.d. = 44.6 ± 14.5 (range 20–78) years.

were all linear. Comparison of the healthy individuals with the cancer patients is invalidated by the age difference between the groups and the age variation of the variables. When the data in Table V are extrapolated to the age of the cancer patients (67 years), the levels of C4 and C1-INH (C4 = 0.39, C1-INH = 0.41) are in between those of the recurrence and no recurrence groups of cancer patients as seen in Table II.

### Discussion

In line with a previous report (Janssen *et al.*, 1989) we found that the preoperative C1-INH and C4 serum concentrations were essential and independent prognosticators of gastric carcinoma. The most significant novel finding in the present study was how the preoperative levels of these variables remained unchanged from before to after surgery and throughout the follow-up period as long as there were no signs of recurrent disease.

When we found that the preoperative C1-INH and C4 levels were independent of the extent of disease, our idea was that they represented a prognostic feature appropriate to the tumour as such or the host in one way or another. As we now have shown that the levels of C1-INH and C4 are also independent of the cancer-bearing state, we suggest that they represent an immune state appropriate to the host. It is a state that indicates whether a resected gastric carcinoma will recur, and also how soon recurrence may be manifest. The prognostic significance of this state, both as to the risk for recurrence and for the time lapse until recurrence, surpasses that of the extent of disease.

One further support for the concept that we are dealing with a state in the host stems from our previous finding among patients who presented with a second primary cancer within 10 years after surgery for gastric carcinoma (Janssen *et al.*, 1990). These patients had the same immune state before gastric cancer surgery as the patients with recurrence of that disease, and in both groups this state was different from that seen in the long time survivors.

A comparison of C1-INH and C4 between the cancer patients and the healthy individuals is uncertain because of the age difference between the groups and the variation of the data with age. It seems, nevertheless, that the age adjusted C4 and C1-INH levels of the healthy individuals are in between those of the two groups of cancer patients. This finding may be due to a general heterogeneity in C1-INH and C4 levels and is in line with our idea that they are tumour independent variables.

Our finding that the preoperative IgG and C4 gave only limited or no prognostic information additional to C1-INH is explained by the correlations between these variables in preoperative sera (Janssen *et al.*, 1983). The prognostic significance of C4 was slightly weaker than that of C1-INH. This may be due to variations of C4 with different histological types of gastric carcinoma (Janssen *et al.*, 1987*b*).

Contrary to C4 and C1-INH, the mean concentrations of IgG were within normal range after surgery and without difference between the groups of patients. This observation indicates that the low levels of IgG preoperatively is a consequence of the tumour-bearing state (Janssen *et al.*, 1987*a*; Tønder & Thunold, 1973; Tønder *et al.*, 1976; 1987). Interestingly, in patients with colorectal carcinoma, IgG varied in the same way as here from the pre- to the postoperative state (Shafir *et al.*, 1980; Bjerkeset *et al.*, 1988).

The time interval from clinical signs of recurrence to death was short in most patients, the median time was 2.5 months. During this period the patients presented a different immune pattern compared to the clinically disease-free period, as previously described (Janssen *et al.*, 1987*c*). Data obtained at or after detection of recurrent disease were therefore not included in the present study.

We think that we are about to identify a state in the host that regulates the growth and spread of gastric cancer. It has been shown that the surface of cancer cells is rich in C1-INH (Osther & Linnemann, 1973; Osther, 1974; Osther *et al.*, 1974). C1-INH may combine with activated C1 and destroy the protease activity and is believed to cause decay dissociation of the C1 molecule. It is now 19 years since these findings were first presented. We are not aware of later significant publications on the biological consequences of C1-INH on the cancer cell surface. We therefore suppose that the effector mechanisms of the immune state that we describe may be sought along other paths. The recorded variations in C1-INH and C4 levels most probably represent 'the top of the iceberg' as expressions of some essential humoral or cellular immune mechanisms.

### References

BJERKESET, T., MATRE, R. & TØNDER, O. (1988). Serum concentrations of IgG, IgA and IgM in patients with adenocarcinoma of the colon and rectum. *Surg. Res. Comm.*, **4**, 103.

GAMEL, J., SEDDON, J., POLIVOGIANIS, L., ALBERT, D. & GREENBERG, R. (1986). A method for assessing potential bias among cancer patients recorded as 'Dead of other causes'. *Cancer*, **57**, 2246.

HERMANEK, P. & SOBIN, L.H. (1987). *TNM Classification of Malignant Tumours.* 4th ed. Berlin, Heidelberg, New York, London, Paris, Tokyo. Springer-Verlag, p. 43.

HOPKINS, A. (1990). Survival analysis with covariates. In: Dixon, W.J. (ed.). *BMDP Statistical Software Manual.* Vol. 2. University of California Press: Berkeley, Los Angeles, Oxford, pp. 769.

JANSSEN, C.W. Jr., TØNDER, O. & MATRE, R. (1983). Stage-related correlations between immunoglobins and complement components in preoperative sera from patients with gastric carcinoma. *Eur. J. Cancer Clin. Oncol.*, **19**, 1601.

JANSSEN, C.W. Jr., MAARTMANN-MOE, H. & LIE, R.T. (1987*a*). Preoperative prediction of extent and prognosis of gastric carcinoma by four serum proteins and erythrocyte sedimentation rate. *Eur. J. Surg. Oncol.*, **13**, 285.

JANSSEN, C.W., Jr., MAARTMANN-MOE, H. & LIE, R.T. (1987*b*). Concentrations of serum proteins and erythrocyte sedimentation rate in patients with different histological types of gastric carcinoma. *Eur. J. Surg. Oncol.*, **13**, 207.

JANSSEN, C.W., Jr., TØNDER, O. & MATRE, R. (1987*c*). Serum concentrations of immunoglobulins and complement components in patients with recurrence after resection of gastric carcinoma. *Cancer Detect. Prevent.*, **10**, 303.

JANSSEN, C.W. Jr., LIE, R.T., MAARTMANN-MOE, H. & MATRE, R. (1989). Serum C1-esterase inhibitor, an essential and independent prognosticator of gastric carcinoma. *Br. J. Cancer*, **60**, 589.

JANSSEN, C.W. Jr., LIE, R.T., MAARTMANN-MOE, H. & MATRE, R. (1990). Who gets a second primary cancer after gastric cancer surgery? *Eur. J. Surg. Oncol.*, **16**, 195.

JENNRICH, R. & SAMPSON, P. (1990). Stepwise discriminant analysis. In Dixon, W.J. (ed.). *BMDP Statistical Software Manual.* Vol.1. University of California Press. Berkeley, Los Angeles, Oxford. p. 339.

LENTNER, C. (1982). *Geigy Scientific Tables.* Vol. 2, 8th (ed.). Basle, Ciba-Geigy Ltd. p. 229.

OSTHER, K. & LINNEMANN, R. (1973). Immunofluorescence measurement of C1 inactivator (alpha 2 neuraminoglycoprotein) activity of the surface of human carcinoma cells. *Acta Pathol. Microbiol. Scand. (B)*, **81**, 365.

OSTHER, K. (1974). C1 inactivator from cancer cells. *Lancet*, **I**, 359.

OSTHER, K., HØJGAARD, K. & DYBKJÆR, E. (1974). Demonstration of a complement inactivator on cultured cells from human malignant brain tumours. *Acta Neurol. Scand.*, **50**, 681.

POSTE, G. & FIDLER, I.J. (1980). The pathogenesis of cancer metastasis. *Nature*, **283**, 139.

SCHLUCHTER, M.M. (1990). Unbalanced repeated measures models with structured covariance matrices. In: Dixon, W.J. (ed.). *BMDP Statistical Software Manual.* Vol. 2 University of California Press: Berkeley, Los Angeles, Oxford, p. 1207.

SHAFIR, M., BEKESI, J.G., PAPATESTAS, A., SLATER, G. & AUFSES, A.H. Jr. (1980). Preoperative and postoperative immunological evaluation of patients with colorectal cancer. *Cancer*, **46**, 700.

TØNDER, O. & THUNOLD, S. (1973). Receptors for immunoglobulin Fc in human malignant tissues. *Scand. J. Immunol.*, **2**, 207.

TØNDER, O., KRISHNAN, E.C., JEWELL, W.R., MORSE, P.A. & HUMPHREY, L.J. (1976). Tumor Fc receptors and tumor-associated immunoglobulins. *Acta Pathol. Microbiol. Scand. (C)*, **84**, 105.

TØNDER, O., MATRE, R. & WESENBERG, F. (1987). Expression and significance of Fc receptors in malignancies. In Kano, K., Mori, S., Sugisaki, T. & Torisu, M. (eds). *Cellular, Molecular and Genetic Approaches to Immunodiagnosis and Immunotherapy.* University of Tokyo Press, p. 135.

WOODRUFF, M. (1982). Interaction of cancer and host. *Br. J. Cancer*, **46**, 313.


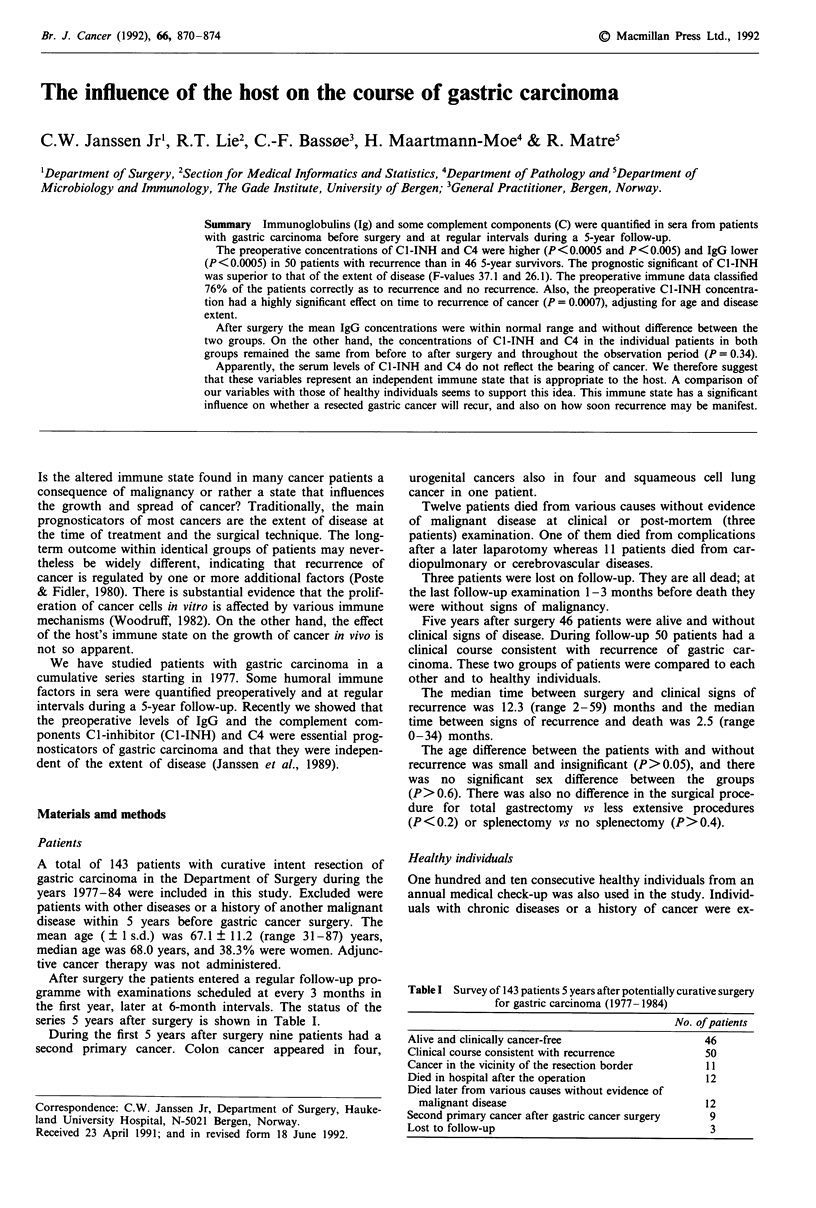

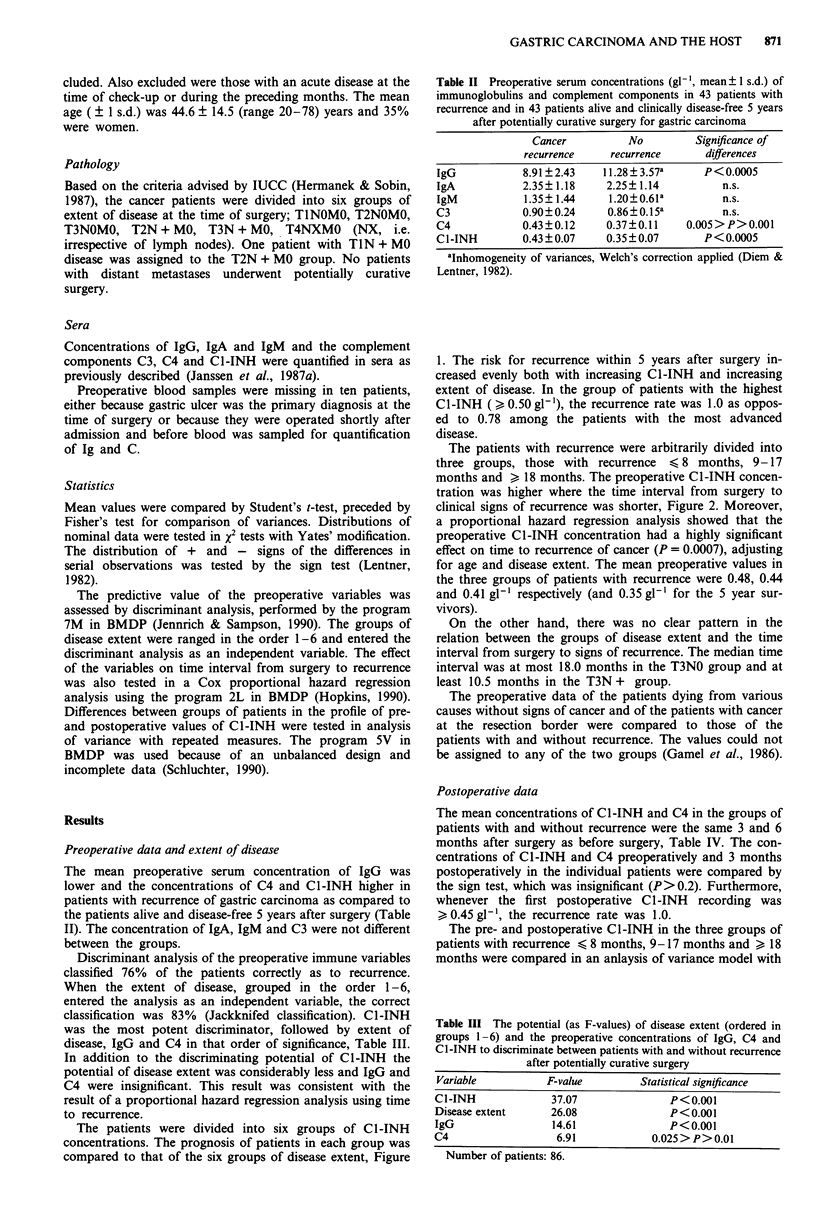

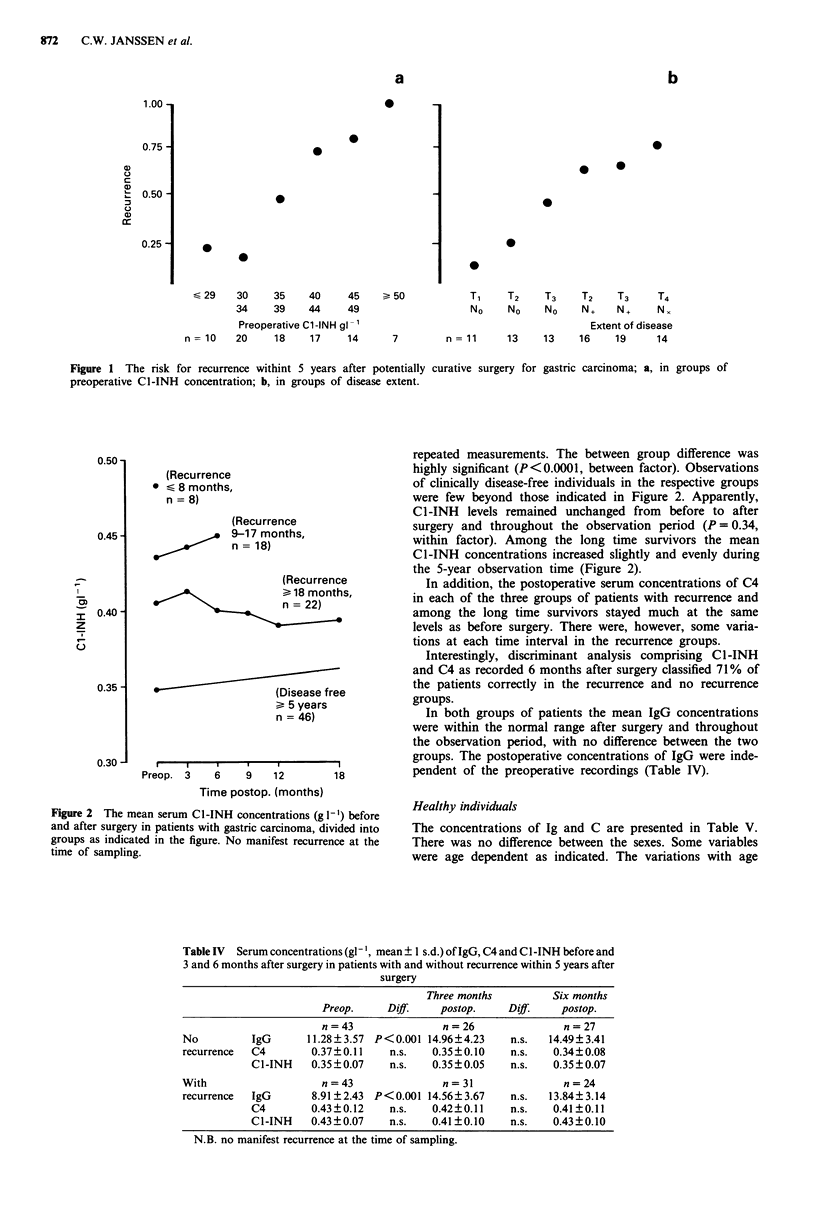

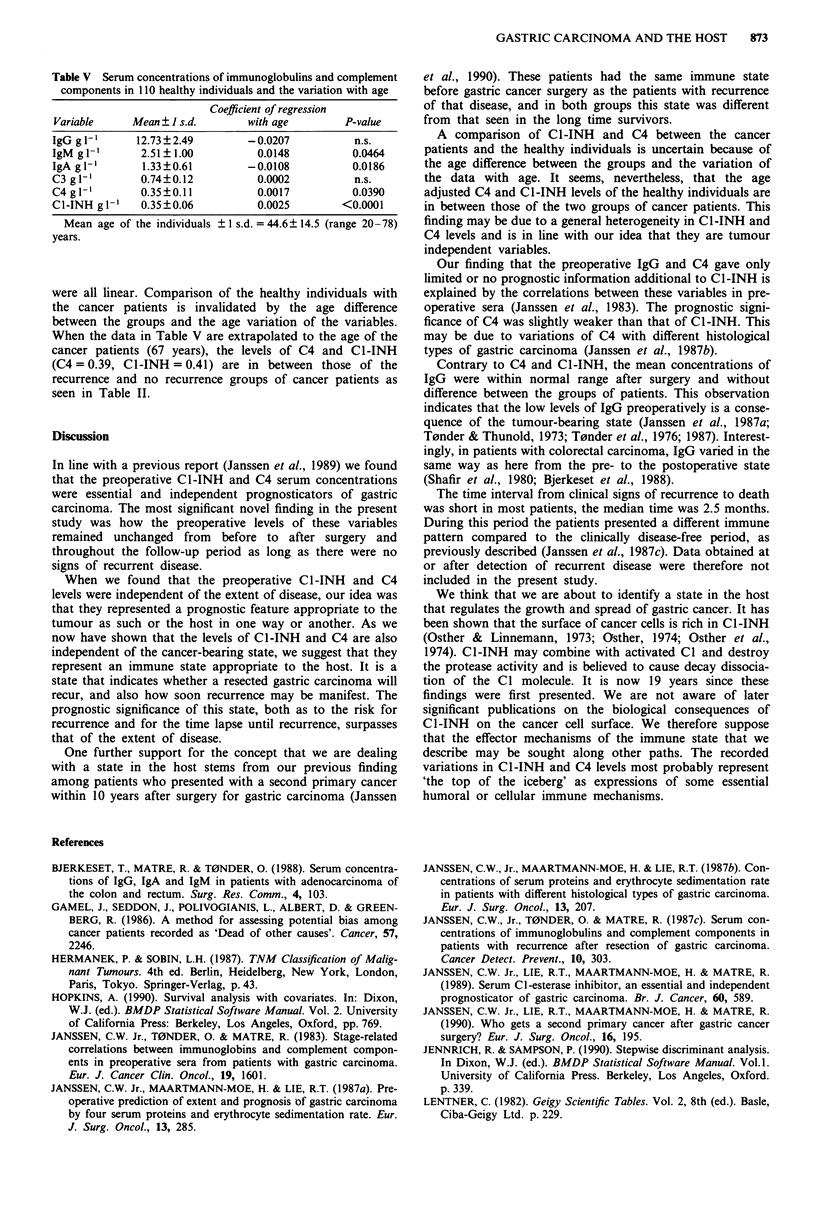

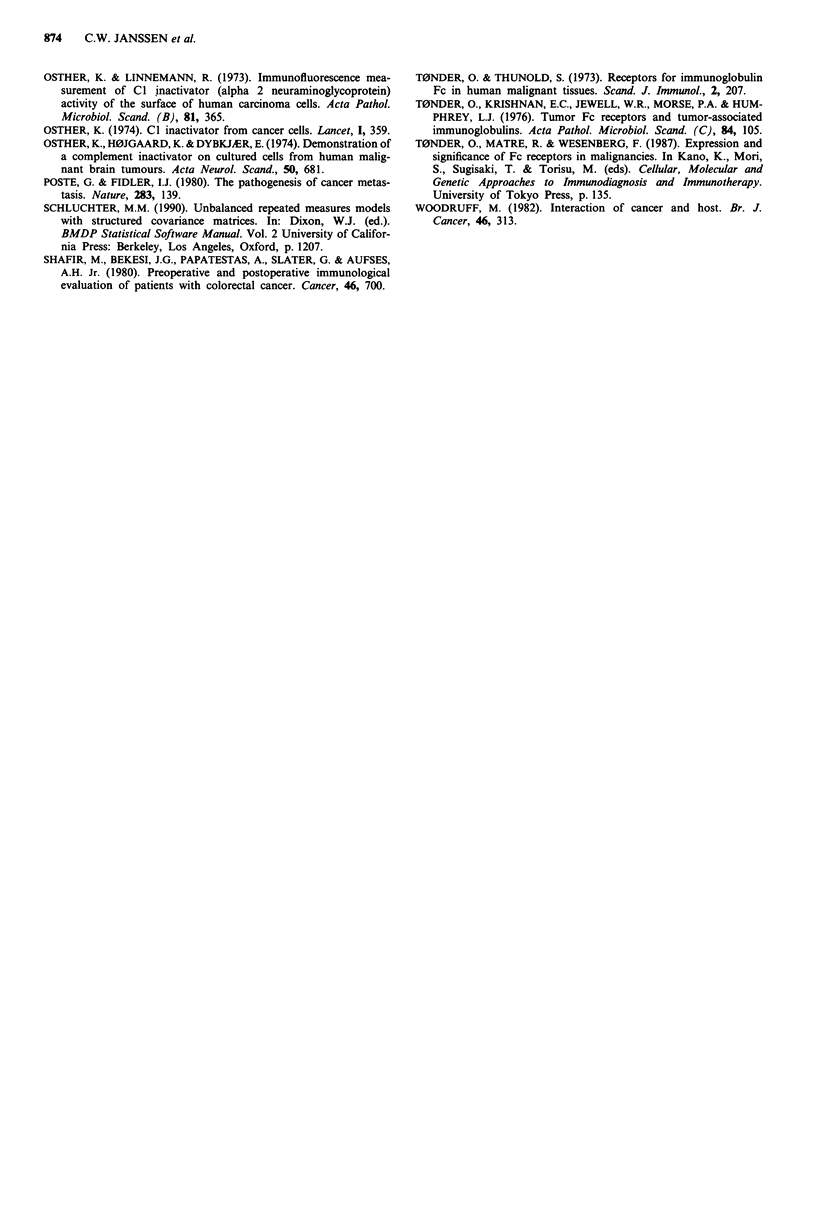


## References

[OCR_00574] Gamel J., Seddon J., Polivogianis L., Albert D., Greenberg R. (1986). A method for assessing potential bias among cancer patients recorded as "dead of other causes." Application to cases of intraocular melanoma.. Cancer.

[OCR_00612] Janssen C. W., Lie R. T., Maartmann-Moe H., Matre R. (1989). Serum C1-esterase inhibitor, an essential and independent prognosticator of gastric carcinoma.. Br J Cancer.

[OCR_00617] Janssen C. W., Lie R. T., Maartmann-Moe H., Matre R. (1990). Who gets a second primary cancer after gastric cancer surgery?. Eur J Surg Oncol.

[OCR_00600] Janssen C. W., Maartmann-Moe H., Lie R. T. (1987). Concentrations of serum proteins and erythrocyte sedimentation rate in patients with different histological types of gastric carcinoma.. Eur J Surg Oncol.

[OCR_00594] Janssen C. W., Maartmann-Moe H., Lie R. T. (1987). Preoperative prediction of extent and prognosis of gastric carcinoma by four serum proteins and erythrocyte sedimentation rate.. Eur J Surg Oncol.

[OCR_00588] Janssen C. W., Tönder O., Matre R. (1983). Stage-related correlations between immunoglobulins and complement components in preoperative sera from patients with gastric carcinoma.. Eur J Cancer Clin Oncol.

[OCR_00606] Janssen C. W., Tønder O., Matre R. (1987). Serum concentrations of immunoglobulins and complement components in patients with recurrence after resection of gastric carcinoma.. Cancer Detect Prev.

[OCR_00641] Osther K., Hojgaard K., Dybkjaer E. (1974). Demonstration of a complement of inactivator on cultured cells from human malignant brain tumours.. Acta Neurol Scand.

[OCR_00640] Osther K. (1974). Letter: C1 inactivation from cancer cells.. Lancet.

[OCR_00634] Osther K., Linnemann R. (1973). Immunofluorescence measurement of C1 inactivator (alpha 2 neuraminoglycoprotein) activity of the surface of human carcinoma cells.. Acta Pathol Microbiol Scand B Microbiol Immunol.

[OCR_00646] Poste G., Fidler I. J. (1980). The pathogenesis of cancer metastasis.. Nature.

[OCR_00656] Shafir M., Bekesi J. G., Papatestas A., Slater G., Aufses A. H. (1980). Preoperative and postoperative immunological evaluation of patients with colorectal cancer.. Cancer.

[OCR_00661] Tonder O., Thunold S. (1973). Receptors for immunoglobulin Fc in human malignant tissues.. Scand J Immunol.

[OCR_00667] Tönder O., Krishnan E. C., Jewell W. R., Morse P. A., Humphrey L. J. (1976). Tumor Fc receptors and tumor-associated immunoglobulins.. Acta Pathol Microbiol Scand C.

[OCR_00676] Woodruff M. (1982). The Walter Hubert Lecture, 1982. Interaction of cancer and host.. Br J Cancer.

